# Active Silver Nanoparticles for Wound Healing

**DOI:** 10.3390/ijms14034817

**Published:** 2013-03-01

**Authors:** Chiara Rigo, Letizia Ferroni, Ilaria Tocco, Marco Roman, Ivan Munivrana, Chiara Gardin, Warren R. L. Cairns, Vincenzo Vindigni, Bruno Azzena, Carlo Barbante, Barbara Zavan

**Affiliations:** 1 Department of Molecular Sciences and Nanosystems, University Ca’ Foscari, Santa Marta, Dorsoduro 2137, 30123 Venice, Italy; E-Mail: chiara.rigo@unive.it; 2 Department of Biomedical Sciences, University of Padova, Viale G. Colombo 3, 35100 Padova, Italy; E-Mails: letizia.ferroni@unipd.it (L.F.); chiara.gardin@unipd.it (C.G.); 3 Burns Centre, Division of Plastic Surgery, Hospital of Padova, via Giustiniani 2, 35128 Padova, Italy; E-Mails: ilaria.toccotussardi@gmail.com (I.T.); ivan.munivrana@unipd.it (I.M.); vincenzo.vindigni@unipd.it (V.V.); bruno.azzena@sanita.padova.it (B.A.); 4 CNR-IDPA c/o Department Environmental Sciences Informatics and Statistics, University Ca’ Foscari, Dorsoduro 2137, 30123 Venezia, Italy; E-Mails: marco.roman@unive.it (M.R.); cairns@unive.it (W.R.L.C.); barbante@unive.it (C.B.)

**Keywords:** silver, nanoparticles, ICP-MS, SEM, TEM, Acticoat™ Flex 3, *in vivo*, *in vitro*, cytotoxicity, mitochondrial toxicity

## Abstract

In this preliminary study, the silver nanoparticle (Ag NP)-based dressing, Acticoat™ Flex 3, has been applied to a 3D fibroblast cell culture *in vitro* and to a real partial thickness burn patient. The *in vitro* results show that Ag NPs greatly reduce mitochondrial activity, while cellular staining techniques show that nuclear integrity is maintained, with no signs of cell death. For the first time, transmission electron microscopy (TEM) and inductively coupled plasma mass spectrometry (ICP-MS) analyses were carried out on skin biopsies taken from a single patient during treatment. The results show that Ag NPs are released as aggregates and are localized in the cytoplasm of fibroblasts. No signs of cell death were observed, and the nanoparticles had different distributions within the cells of the upper and lower dermis. Depth profiles of the Ag concentrations were determined along the skin biopsies. In the healed sample, most of the silver remained in the surface layers, whereas in the unhealed sample, the silver penetrated more deeply. The Ag concentrations in the cell cultures were also determined. Clinical observations and experimental data collected here are consistent with previously published articles and support the safety of Ag NP-based dressing in wound treatment.

## 1. Introduction

For centuries, silver compounds and ions have been extensively used for both hygienic and healing purposes, due to their strong bactericidal effects, as well as a broad spectrum antimicrobial activity [1,2]. Taking advantage of its bactericidal properties, various silver-containing preparations have been used for the treatment of chronic wounds. In the 17th and 18th centuries, silver nitrate was already used for ulcer treatment, and in 1960, it was introduced for the management of burns. After a decrease in the use of silver salts consequent to the introduction of antibiotics in 1940, in more recent years, there has been a renewed interest in silver, due to increased resistance of bacteria to antibiotics and improvements in polymer technology. This has resulted in a large number of silver-containing dressings being available on the market. Silver is applied to burns, either in the form of impregnated bandages or as a cream containing silver sulfadiazine as the active agent, a product that is still considered the benchmark silver product [3]. At the end of the 1990s, several Ag-containing dressings from different manufacturers appeared in commerce. Silver-based dressings are now available as a variety of fibers or polymeric scaffolds impregnated or coated with a Ag salt or metallic Ag in nanoparticulate form. They all exhibit fast and broad spectrum antibacterial activity against both Gram-positive and -negative bacteria [4,5]. In recent years, the mechanism of action of silver has been investigated: it seems that silver shows a multilevel antibacterial effect, due to blockage of respiratory enzyme pathways, as well as alteration of microbial DNA and the cell wall [6]. Silver has been demonstrated to be effective also against multidrug-resistant organisms [7,8], whilst maintaining a low systemic toxicity [9]. Clinically, several studies have confirmed their safety for patients [10,11] and concerns about their cytotoxicity on fibroblasts and keratinocytes have not been confirmed [12–14].

Nanoparticles (NPs) are defined as particles having one or more dimensions in the order of 100 nm or less. Silver NPs (Ag NPs) have been shown to possess unusual physical, chemical and biological properties [15–17]. The effectiveness of Ag NP-containing dressings has been widely tested *in vitro*, and much research work has been published recently demonstrating that these dressings have a fast and broad spectrum antibacterial activity against both Gram-positive and -negative bacteria [18,19]. However, up to now, the number of *in vivo* studies has been limited [20,21]. Even though Ag NPs-containing dressings are declared to be safe for patients and non-cytotoxic [22–24], recent studies have shown possible toxic effects on human fibroblasts and keratinocytes [25,26]. The cytotoxic effects that have been observed in different cell lines *in vitro* include decreases in mitochondrial function [27], cellular shrinkage and irregular shape [28], as well as production of reactive oxygen species (ROS) [29,30]. Carlson *et al.* found that ROS production was particle size, as well as concentration dependent [31], whilst Hackenberg and co-workers found that Ag NPs induce DNA damage in human mesenchymal stem cells [32].

Considering the effective antibacterial properties of Ag NPs and the enormous interest in their application as coatings for medical devices and in wound therapy, their safety and biocompatibility need to be urgently clarified. We present here an in-depth study of a Ag NPs containing dressing. The product chosen is a flexible polyethylene cloth coated with nanocrystalline Ag particles and is one of the most widely used Ag based dressings in burns centers worldwide. The dressing was developed to guarantee a controlled and prolonged release of nanocrystalline silver [33] to the wound area; according to the manufacturer, the nanosilver particles also release silver ions. Physical vapor deposition was used to coat the polyethylene with nanocrystals that have a mean diameter of 10–15 nm [34–36]. Previously published *in vitro* studies regarding the safety of this product have been carried out on human derived skin cells [37,38] and are in contrast with *in vivo* studies [39,40] and with daily observation of patients treated with this dressing [41].

In an attempt to clarify matters, our research work was carried out *in vitro* on a three dimensional cell culture system of human skin fibroblasts [42,43], as well as *in vivo* on skin biopsies from burns patients. The dressing was applied onto the cells and was changed every three days to simulate dressing changes by a clinician. The Ag concentration in the culture medium and in the used dressings has been measured by inductively coupled plasma mass spectrometry (ICP-MS), and the Ag amount absorbed by the cells has been evaluated by difference. Biochemical and morphological techniques have been employed to determine both the viability and the spatial distribution of the cells during the application of the dressing. In order to compare *in vitro* with *in vivo* results, skin biopsies were taken from a patient at different times during the healing process. These samples were subjected to histological analysis, and transmission electron microscopy (TEM) analyses were performed to determine the distribution profile of Ag NPs and their subcellular localization.

Our *in vitro* results confirm that Ag NPs alter mitochondrial functionality in human fibroblasts, but interestingly, this does not seem to lead to cell death. Despite the reduced metabolic activity, we demonstrate that the cells are still viable, as no features of apoptosis were detected. We retain that the apparent cytotoxicity observed by other authors is probably an artifact of the use of a mitochondrial specific activity assay that is an indirect measure of cells viability.

Our results suggest that mitochondria are activated to protect the cell and, in particular, the nucleus, against the action of Ag NPs. *In vivo* results demonstrate that application of Ag NP-based dressings allows wound healing and recovery. For the first time, it has been demonstrated that during application of Ag NP-based dressings on real patients, Ag NPs are released and enter into the cells as agglomerates. The Ag NPs do not dissolve entirely, but remain in the fibroblasts’ cytoplasm during the whole healing process and change shape over time. This effectively demonstrates the safety of Acticoat™ Flex 3, a Ag NP-based product currently used in burn care. Although Ag NPs treatment reduces mitochondrial activity, it does not appear to affect cell viability. Hence the Ag NPs released could be defined as toxic to mitochondria, causing a temporary reduction in metabolic activity in the cell, without causing cell death. Cells remain viable and are able to re-proliferate once the silver is passivated, leading to reconstruction of the dermal tissue *in vivo*.

## 2. Results and Discussion

### 2.1. *In Vitro* Study

#### 2.1.1. MTT Assay

To investigate if Ag NPs could negatively affect the healing process, we evaluated their toxicity on fibroblasts *in vitro*. A collagen-based scaffold was employed as a support for a 3D cell culture of fibroblasts to obtain a dermal-like tissue. At three, six and nine days, MTT assays were carried out to assess the mitochondrial function in cells treated with Ag NPs.

**Figure 1:**
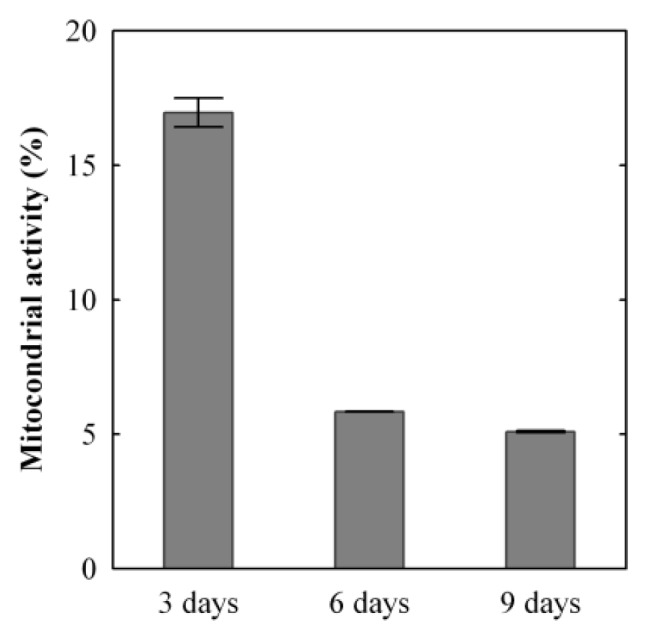
Mitochondrial activity in silver nanoparticle (Ag NP)-treated 3D fibroblast cultures, (one sample *n* = 2 readings ± SD *versus* time). The mitochondrial activity in the treated samples is expressed as a percentage of the activity of the untreated samples. For each time point, MTT values were obtained from duplicate readings of a single sample.

As reported in Figure 1, a time-dependent decrease in metabolic activity was observed in the cells treated with the Ag NP-based dressing. This confirms the ability of Ag NPs to impair mitochondrial function, as reported by Burd [12] and Foldbjerg [27].

Our results demonstrate that the mitochondrial activity rapidly decreases over the first three days of treatment. After three days of exposure to Ag NPs, the cells had only 17% ± 0.54% of mitochondrial functionality normalized to the untreated sample value. In the following three days, the relative mitochondrial activity decreased to 7% ± 0.01%. In the final three days of the experiment, the mitochondrial functionality relative to the untreated control dropped to 5% ± 0.03%. This indicates that the Ag NPs and silver ions heavily impair mitochondrial functionality, and this is probably correlated to the generation of ROS, in agreement with the results found by AshaRani [28] and Hsin [30].

#### 2.1.2. Morphological Analysis

**Figure 2:**
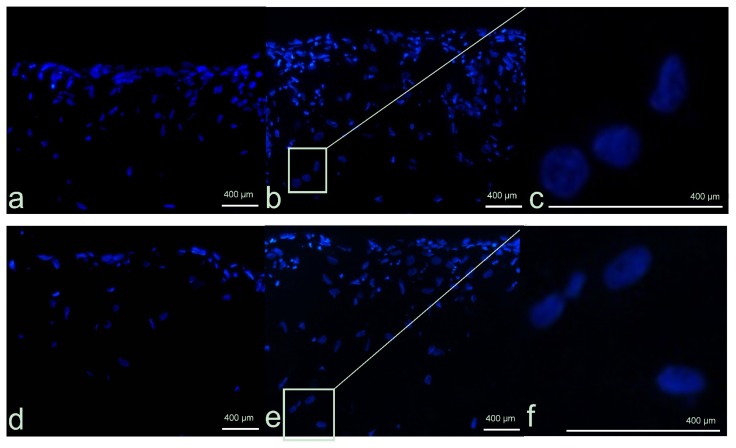
Dermal-like tissue reconstructed *in vitro*. Cells, visible thanks to the Hoechst blue staining of the nuclei, can be seen inside the collagen-based scaffold and appear to be organized in layers. (**a**) Un-treated control after three days from the beginning of the experiments; (**b**) Untreated control at nine days; (**c**) Enlargement of selected area; (**d**) Ag NP-treated fibroblast at three days; (**e**) Ag NPs fibroblast at nine days; (**f**) Enlargement of selected area.

Morphological analyses were carried out to investigate nuclei morphology and cellular distribution within the scaffold. Hoechst dye was employed to stain the nuclei blue and was used to verify the nuclear integrity or the presence of any apoptotic features, such as chromatin condensation and fragmentation, as well as the presence of apoptotic bodies. Under UV light, the characteristic fluorescence at 461 nm allows the localization of the cells in the three dimensional matrix. To confirm the results obtained, YO-PRO®-1 staining was carried out. As reported in Figure 2a, in the untreated 3D fibroblast cultures (controls), the cells proliferated mainly on the surface, although some cells grew also inside. Therefore, the dermal-like tissue appears as a multilayer of cells, where the fibroblasts are able to proliferate during the course of the experiments (Figure 2b). No signs of apoptosis were detected (Figure 2c).

The similar distribution of cells was seen in the Ag NP-treated samples (Figure 2d). Interestingly, despite the reduced mitochondrial functionality observed, the nuclei are still present and appear to be undamaged. There was no observable presence of apoptotic bodies or nuclear fragmentation (Figure 2e,f). These results are in agreement with those recently observed by Zanette *et al.*[26] in the HaCaT cell line, supporting the hypothesis that Ag NPs reduce mitochondrial functionality without seeming to cause genotoxicity and cell death.

**Figure 3:**
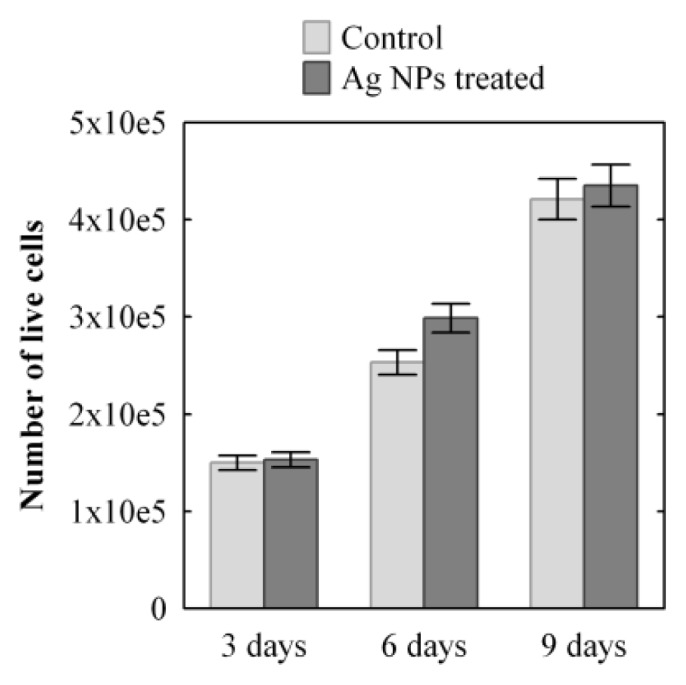
Progression of cell growth in time in a 3D dermal-like tissue after Ag NPs treatment (dark grey) and in the control sample (light grey); mean value ± SD (*n* = 2) samples *versus* time. The count of the live cells in the sample is obtained as the sum of the live cells at various depths at each position.

In Figure 3, a quantitative comparison of the number of live cells in the treated and untreated 3D cell cultures can be seen. The results show that the number of live cells increased with time at the same rate in both samples. The YO-PRO®-1 assay showed that there were no apoptotic cells visible in the sample treated with Ag NPs.

#### 2.1.3. Ag Release and Distribution

The level of Ag in the new unused dressings was 821 ± 20 μg cm^−2^ (*n* = 9). This result is in agreement with the value of 827 ± 58 μg cm^−2^ previously estimated by Rigo *et al*. [44] and demonstrates that the inter-batch variability is negligible for Acticoat™ Flex 3. Table 1 summarizes the concentrations of Ag, and it is expressed as μg of Ag per cm^2^ (μg cm^−2^) of culture covered with the dressing. This is reported as per individual application of the dressing. During the experiment, three consecutive applications of Ag NP-based dressing were carried out. For the second and third application, the old dressing was substituted with a new one and deposited on the same sample of the cell culture. Concurrently, the medium (cDMEM) was removed and substituted with the same volume of fresh medium. The level of Ag applied to the culture at each step was derived from the average concentration of the unused dressing (see above), considering that all the pieces applied had the same surface area (0.283 cm^2^, 6 mm diameter). The fraction corresponding to the used dressing (called Dressing in Table 1) is comprised of Ag that was never released and Ag that was released, but re-adhered to the surface in another form, e.g., small fragments of scaffold containing Ag stuck to the surface. The fraction medium represents the Ag released into the liquid medium, and the fraction culture was calculated by difference and represents the Ag captured by the 3D culture.

For each individual application of the dressing, most of the Ag (~94%) was revealed to remain in the dressing. The result is compatible with those previously obtained for the release of Ag in solution [44], where 94%–99% of Ag was found to still be present in the dressing after three days, depending on the composition of the solution. In this study, the fraction with the smallest silver concentration is the cell culture. Although the concentration is low, it is not irrelevant: the cumulative value reaches 68 ± 7 μg cm^−2^ (*n* = 3) after nine days of treatment. Although the cell culture was not changed at each three-days (unlike the dressing and the medium), the absorption rate of Ag increased from 18 ± 3 μg cm^−2^ (*n* = 9) in the first step to 25 ± 2 μg cm^−2^ (*n* = 6) in the second and 25 ± 3 μg cm^−2^ (*n* = 3) in the third steps. Such an inverse trend between Ag and relative mitochondrial activity (see Figure 1) levels in the culture seems to confirm their direct causal relationship. The fraction of Ag released in the medium follows an opposite trend with decreasing values from the first to the second step. This can be explained by the fact that the dressing releases a constant quantity of Ag into the medium, but cellular uptake increases in the second and third steps. In order to validate the indirect estimates of Ag concentration in the cell cultures reported in Table 1, the Ag concentrations in the samples used for the MTT analyses were determined by ICP-MS by analyzing the MTT and iDMSO fractions. These direct measurements and their sum are reported below in Table 2 and are compared with the estimated results obtained above.

The direct determination of Ag in the cell cultures resulted in levels lower than the indirect estimates, which are derived from the mass balance of the metal. It should be noted that the direct determinations are based on single replicates, so an uncertainty value cannot be provided. The underestimation is probably due to a non-quantitative recovery of Ag by the two extractions (in MTT buffer and iDMSO), so that part of Ag probably remains bound to the scaffold. We have shown that the accurate direct determination of Ag in the culture would require a separate replicate of the experiment. The indirect determination of Ag is a viable alternative to describe the kinetics of Ag distribution in the system, reducing the amount of time and materials required. The fraction extracted into iDMSO is always greater than that in MTT, and the proportion increases almost linearly between the third to the ninth day of treatment.

In the control experiment, the Ag amount measured in the MTT fraction is 70% of the total and is similar to the Ag concentration found in the MTT fraction after six days, when fibroblasts were present. The amount of Ag measured in the iDMSO fraction is 25% of the Ag released and is much less than found when cells are present, and the amount of Ag found after dissolution of the scaffold was found to be 5% of the total. This partitioning can be easily explained, because MatriDerm® is a dermal substitute made of elastin and collagen type I, III and V obtained from bovine nuchal ligaments (as stated by the manufacturer); these proteins have a limited number of cysteine residues. Therefore, Ag binds loosely to MatriDerm® and is easily extracted during the first extraction (MTT fraction). The Ag concentration in the MTT fraction should represent the amount of Ag loosely bound to the scaffold or the external surfaces of the cell membranes. It is well known that Ag has a strong interaction with DMSO [45] and that it causes cell lysis. So, it can be reasonably assumed that the Ag concentration in the iDMSO fraction represents the major part of the Ag fraction inside the cells, as well as being strongly bound to the structural constituents of the cells. This is confirmed by the control experiment that shows that the amount of Ag extracted by the iDMSO solution from the scaffold is always much less the value found when cells are present, and that complete dissolution of the scaffold only releases 5% of the total Ag present. This demonstrates that most of the Ag released from the dressing over the nine days of the study is taken up by the cells or is adsorbed strongly to their surface. Considering that there is a linear increase of Ag in the iDMSO fraction during the experiment, we conclude that Ag uptake by the cells did not cease and that the cellular Ag binding sites either in or outside the cells had not been saturated.

### 2.2. *In Vivo* Study

#### 2.2.1. Microscopy

**Figure 4:**
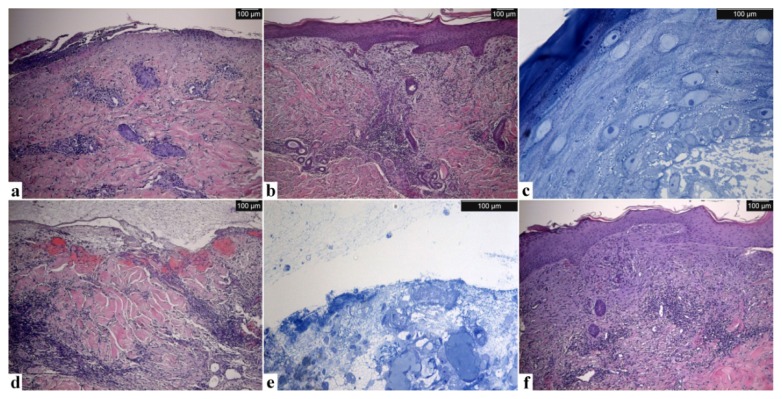
Optical microscopy (OM) Images of the skin samples: burnt (**a**), healed after seven days (**b** and **c**), unhealed after seven days (**d** and **e**) and after complete re-epithelialization (**f**) after 10 more days of treatment. Healed and unhealed skin sections are shown with H/E and toluidine blue staining. Scale bar: 100 μm.

Optical microscopy observations of the skin biopsies after hematoxylin eosin staining show the main differences between the four samples. Figure 4a is burnt skin after surgical cleaning before silver application. Figure 4b is healed skin after seven days of treatment. Figure 4d is unhealed skin after seven days of treatment, and Figure 4f shows the same area healed after 17 days of treatment.

The burnt skin prior to Ag NP dressing application shows epidermal necrosis, diffuse perivascular infiltrate and important collagen degeneration at the level of the papillary dermis, characteristic of a deep dermal burn (Figure 4a).

After seven days of treatment with Acticoat™ Flex 3, the structure of the biological tissue has been completely restored in the healed area (Figure 4b). Under staining with toluidine blue, it can be seen that the healed skin is composed of a well-stratified epidermis, complete with basal, spinous, granular and cornified layers (Figure 4c). Macroscopic evaluation shows more over the presence of a well vascularization at the dermal–subcutaneous interface. In the unhealed skin samples (Figure 4d,e), the tissue organization has not been re-established. The epidermis has not yet re-formed (Figure 4d) and the dermis still has a disorganized and irregular structure (Figure 4e). The wound was treated with Acticoat™ Flex 3 for 10 more days. When the dressing was removed, the wound appeared healed, and a new biopsy was taken to verify the re-establishment of the tissue architecture. Optical microscopy observations of the sample (Figure 4f) confirm the re-growth of the tissue structure, and in particular, it is possible to observe the restoration of the epidermis. This result of complete patient healing by the 17th day shows that, despite the presence of Ag NPs in the tissue and inside the cells, the healing process does not seem to be impeded. Our results agree with and support the results recently published by Gravante *et al.*[46]. They found that Acticoat™ Flex 3 was the dressing with the shortest healing times for deep partial thickness burns (16 days average; our result, 17 days). The average healing times for sodium carboxymethyl cellulose were longer than those of nanocrystalline silver (21 days), but were shorter than paraffin gauzes (26.5 days) and collagenase cream (29 days). This data confirms that the healing process is not impeded during the treatment with Ag NPs.

**Figure 5:**
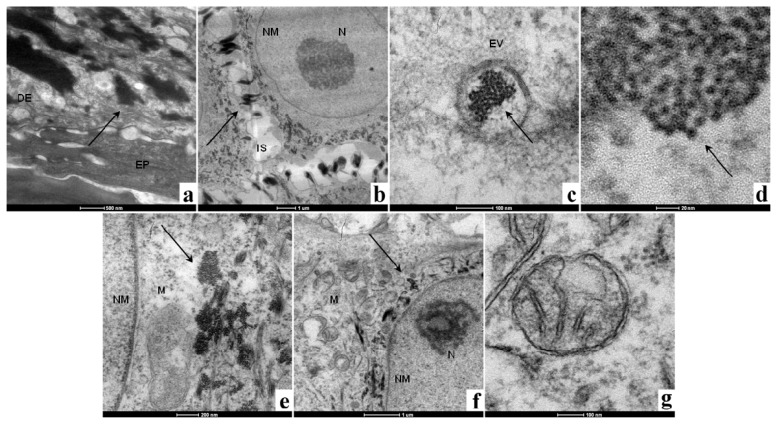
TEM images of the healed skin sample. (**a**) epidermis and dermis; (**b**) detail of a fibroblast surrounded by Ag NPs, in the upper part of the dermis; (**c**) endocytic vesicle containing Ag NP agglomerates; (**d**) magnification of endocytic vesicle containing Ag NP agglomerates; (**e**) Ag NPs have been released into the cytoplasm of a fibroblast and are located near the mitochondria; (**f**) a fibroblast in the lower part of the dermis—Ag NP aggregates are near the nuclear membrane; (**g**) a healthy undamaged mitochondrion. Key: DE, dermis; EP, epidermis; EV, endocytic vesicle; IS, intercellular space; M, mitochondrion; N, nucleolus; NM, nuclear membrane. Arrows indicate Ag NP agglomerates. Scale bars: (**a**) 500 nm; (**b**) 1 μm; (**c**) 100 nm; (**d**) 20 nm; (**e**) 200 nm; (**f**) 1 μm; (**g**) 100 nm.

TEM images of the healed skin sample after seven days are shown in (Figure 5a–g). The sample was observed from the epidermis to a point at which Ag NPs were no longer visible, which corresponded to a depth of ~3 mm. In Figure 5a, the presence of a great number of agglomerates of nanoparticles can be seen surrounding the fibroblasts in the upper part of the dermis. No Ag NPs or aggregates were visible in epidermis. A higher magnification of the same area shows that the aggregates are located in the extracellular matrix, close to the cell membrane. In Figure 5b, as described by Xu [47], a slight broadening of the intercellular space can occur and is probably due to a previous inflammatory phase. In Figure 5c, it can be seen that agglomerates of Ag NPs enter into the cells via endocytic vesicles in the form of agglomerates, as recently observed separately by Kim [48] and Greulich [49] in different cell lines. The individual nanoparticles that are visible were measured to have diameters of <10 nm (Figure 5d). After the agglomerates were released into the cytoplasm (Figure 5e), they typically had a round shape and were located close to the mitochondria (labeled M). No Ag NPs were detected inside the nucleus (labeled NM), and no fragmented nuclei were observed. Despite the presence of Ag NPs inside the cytoplasm, the nuclear membrane is intact and is round in shape. The nucleolus visible in Figure 5b (labeled N) confirms that the chromatin is not condensed, but that it has a structure that allows transcription to proceed. In Figure 5e, it was observed that the mitochondria are generally located in the proximity of the nuclear membrane.

It was observed that the fibroblasts in the upper part of the dermis seem to possess a particular spatial distribution of mitochondria. We speculate that these organelles have been replicated and then moved by the microtubules [50,51] to surround the nucleus to protect the DNA from possible damage caused by Ag NPs. It has been already demonstrated in different cell lines that Ag NPs are capable of generating ROS inside the cells [29,31] that could damage the genetic material [32]. We hypothesize that once the Ag NPs have been released into the cytoplasm, they generate ROS. This could result in an increase in the number of mitochondria as a response to oxidative stress, as demonstrated by Lee *et al.*[52], to try and compensate for the reduced activity, as seen in Figure 3. We speculate that mitochondria are moved by the microtubules around the nucleus to act as a physical, as well as “chemical”, barrier to prevent ROS and Ag NPs from reaching the nuclear membrane. If the mitochondrial membrane breaks down due to the action of ROS, antioxidative enzymes, such as mitochondrial superoxide dismutase (mtSOD), catalase, glutathione peroxidase and thioredoxin peroxidase [53], are released from the mitochondria in the cytoplasm to quench the ROS. It has been already demonstrated that Ag NPs and Ag ions cause increased levels of SOD in human [54] and yeast [55] cells and that *in vitro*, the administration of ROS scavengers (such as SOD, catalase, mannitol and sodium selenite) can partially block the genotoxic effects of Ag NPs in human bronchial epithelial cells [54].

In the lower part of the dermis (Figure 5f,g), the Ag NP agglomerates have a different shape and location inside the fibroblast, compared to what has been found in the upper stratus of the dermis. The agglomerates appear elongated and are located very close to the nuclear membrane. The mitochondria (labeled M) are generally distant from the agglomerates, indicated with an arrow, and appear to be undamaged and healthy (Figure 5g).

As Acticoat™ Flex 3 was applied to the surface of the burn, the cells in the wound bed (lower part of the dermis) were the first to receive the Ag NPs released from the dressing. These cells were exposed to Ag NP aggregates for a longer time with respect to fibroblasts of the upper part of the dermis. During this time period, the Ag NPs could have undergone chemical changes that passivated them or quenched their ROS generating ability. In light of this hypothesis, Ag NPs could remain inside the cells, without provoking toxic effects. This could explain the different spatial distribution of the Ag NP agglomerates in the fibroblasts of the upper and lower dermis, but further investigations are necessary.

**Figure 6:**
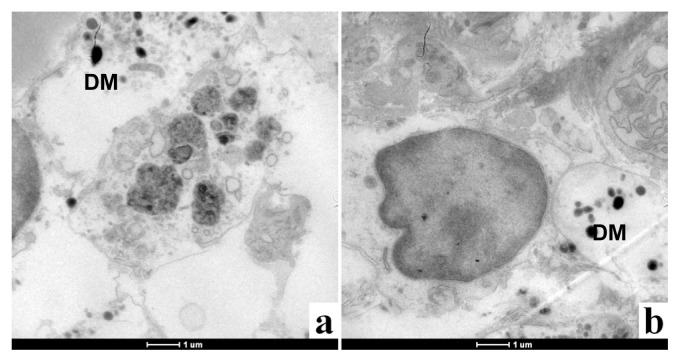
TEM images of the unhealed skin sample, DM (dark matter), contained in vesicle. Scale bar: 1 μm.

In the unhealed skin, the tissue structure is highly disorganized and the epidermis had not reformed. The TEM images in Figure 6a,b show vesicles containing electron-dense matter, but it was not possible to verify if this material contained Ag. Unlike in the healed skin, in the unhealed skin sample, it was not possible to identify nanoparticles. It is possible that the electron-dense matter is composed of Ag salts or Ag bound to proteins via thiol or selenide groups and then precipitated [56].

**Figure 7:**
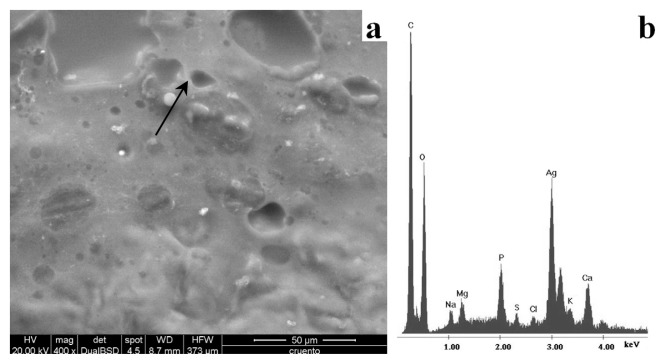
SEM images of the unhealed sample (**a**) and energy-dispersive X-ray spectroscopy (EDS) spectrum (**b**) of the particle indicated by the arrow. Scale bar: 50 μm.

The SEM analyses showed the presence of particles in the unhealed skin samples. As shown in Figure 7a, such particles have a diameter <10 μm. The EDS spectrum (Figure 7b) shows that the particles are composed of virtually pure Ag metal. In the healed skin sample, it was not possible to identify Ag in any form. This was probably due to the low sensitivity of the technique and the absence of Ag microparticles on the surface of the sample. However, Ag microparticles were identified by SEM-EDS in the skin sample collected after 17 days of treatment, when the previously unhealed part of the wound had returned to a healthy condition.

#### 2.2.2. Ag Release and Depth Profiles

**Figure 8:**
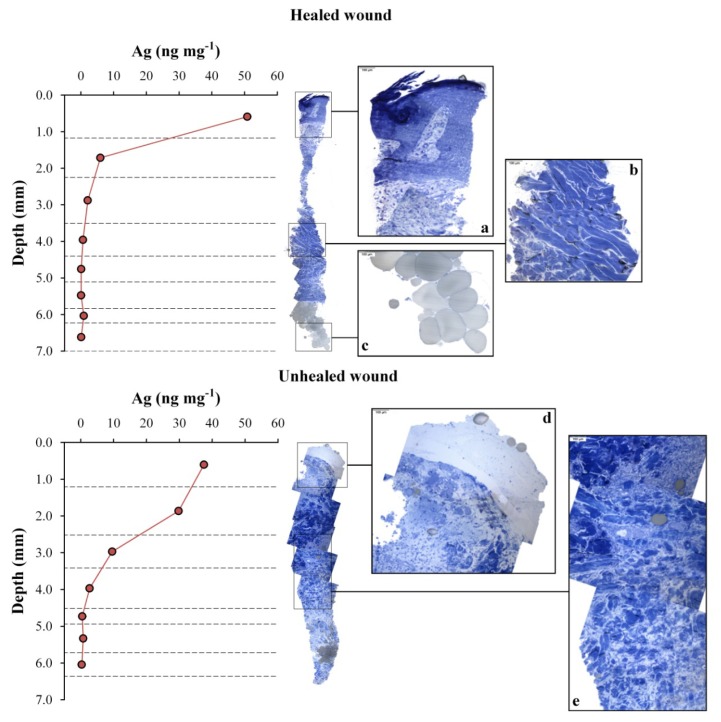
Depth profiles of Ag concentration (ng mg^−1^) after seven days of treatment. In this pilot study, the biopsies were obtained from the same patient. One biopsy for silver analysis was taken from the healed part of the wound (**top**) and one from the unhealed (**bottom**) tissue. Panoramic images of the other duplicate sample were obtained by optical microscopy, and zoomed in areas of representative portions are also shown for comparison.

The depth profiles of Ag concentrations in the healed and unhealed biopsies taken after seven days of treatment are shown in Figure 8. This shows that the level of Ag in the healed sample decreases rapidly from 50.8 ± 0.8 ng mg^−1^ to 6.0 ± 0.2 ng mg^−1^ between the first and second slices. This trend continues between the third and deepest slices, to reach a range between 0.04 and 0.1 ng mg^−1^. The superficial concentration of Ag in the unhealed sample after seven days of treatment was lower (37.5 ± 0.6 ng mg^−1^), but the drop in the concentration profile was more gradual, as the second and third slices still had high concentrations of the metal (29.8 ± 0.5 and 9.6 ± 0.2 ng mg^−1^, respectively) compared to the healed sample. The cumulative amount of Ag in the unhealed tissue was 223 ng, which is slightly higher than the value in the healed tissue of 171 ng. The sample that was collected after an additional 10 days of treatment had a Ag profile (not shown) similar to that of the healed tissue after seven-days of treatment, but with approximately double the concentrations: 110 ± 2, 31.0 ± 0.7 and 13.2 ± 0.1 ng mg^−1^ in the first three slices, respectively.

The comparison between the Ag profiles and the corresponding OM images of the biopsies is also shown in Figure 8. This allows us to correlate the penetration of the metal into the tissue with its structural organization. The healed skin sample has the organized structure of a well-reconstructed tissue. The Ag NP aggregates released from the dressing were not able to penetrate deeply into the tissue, so they remained immobilized, mainly in the upper stratum of dermis, where they were covered by newly grown keratinocytes. The unhealed skin, instead, has a messy and disorderly structure and the normal connective tissue organization has not yet been re-established. Fibroblasts are low in number and the connective tissue poorly formed. This means that the Ag NPs released by Acticoat™ Flex 3 are able to pass through the connective tissue to reach the lower strata of the dermis. As observed in the quantification of Ag in the *in vitro* MTT and iDMSO fractions (see above, Section 2.1.3), the Ag concentration in the skin samples increases linearly with the number of dressings applied on the wound. The same is seen *in vivo*; a skin sample treated with two pieces of Acticoat™ Flex 3 has double the Ag concentration of skin samples after only one application. The slightly higher cumulative Ag concentration measured in the unhealed skin sample after seven days of treatment can be explained by considering that the presence of body fluids and exudates can increase Ag release from dressings [56].

#### 2.2.3. Clinical Observations

Our results are reinforced by the clinical observations that were collected between January 2011 to September 2012 in the Plastic Reconstructive Surgery Division. In this time interval, 98 patients were treated with Acticoat™ Flex 3 for coverage of burn wounds. A total of 102 applications, corresponding to 58,400 cm^2^ of dressing containing a total of nearly 48 g of high purity silver, were used. The annual use in 2011, which was the year of introduction of Acticoat™ Flex 3, was around 33,000 cm^2^. Data from average monthly use in 2012 are comparable to 2011, and we foresee a stable annual use. The patients were 63 males and 45 females, with an average age of 49 years. Ninety-five patients received a single silver dressing application; three patients, two. Burning causes comprised of fire (55 patients), hot liquids (23, of which three were boiling oil) and others (six contact burns, four chemical burns). The average total body surface area (TBSA) burn percent was 23.6% ± 17.1%. Out of 98 cases, 100% reached re-epithelialization. The average amount of dressing used per patient was 602.4 ± 163.5 cm^2^ (minimum 200 cm^2^; maximum 1100 cm^2^). Re-epithelialization was spontaneous in 80 cases, and lesions were recorded as healed within 32 ± 18 days from the last silver dressing application. None of the patients in this study complained of pain or had any symptoms or signs suggesting argyria. None of the 98 patients treated with Acticoat™ Flex 3 between January 2011 to September 2012 in the Plastic Reconstructive Surgery Division was recorded to have staining of skin due to silver accumulation. Eighteen patients reached healing after autologous skin grafting. Autologous grafting was performed at 23 ± 9 days after the last silver dressing application. Healing times recorded for burn wounds are consistent with the data previously reported by other authors [34,57,58]. Khundkar *et al.*[20] demonstrated that Ag NP-based dressings are effective in reducing the time for re-epithelialization and the requirement for grafting in comparison to other treatment widely used in burn centers, such as silver sulfadiazine and 0.01% neomycin and polymyxin solution. Strand *et al.*[59] compared the length of stay for hospitalized patients treated with Acticoat™ Flex 3 with those treated with Mepitel, a flexible polyamide net coated with soft silicone. The authors found the mean in-patient stay was 12.5 days when treated with Mepitel, whilst the mean stay was 4.5 days when the patients were treated with nanocrystalline silver dressing. In our study, the percentage of patients (18%) that required skin grafting is limited and comparable to the results (8%) obtained by Strand *et al.*[59]. Literature data, clinical observations collected by the Plastic Reconstructive Surgery Division of Padua and the results obtained in this work on a limited number of samples support each other in confirming the safety of Acticoat™ Flex 3.

#### 2.2.4. Limits of This Study and Future Perspectives

A major limit of this preliminary work is the number of *in vitro* and *in vivo* samples that were made available. In particular, it would be useful to verify the results of the MTT and YO-PRO®-1 assays on a larger number of samples and recruit more patients for the study. Another limit of this work is that the mitochondrial activity was only monitored for nine days, which was not long enough to verify the revival of mitochondrial activity that is suggested by our *in vivo* observations. To clarify this aspect, the *in vitro* experiments should be carried out for a prolonged time period (>17 days, time for complete healing in patients). The side effects and fate of Ag NPs remaining deposited in the healed skin after treatment are still unknown and should be investigated by patient follow-up in any future work.

## 3 Material and Methods

### 3.1. Dressing

Acticoat™ Flex 3 (Smith & Nephew, Milan, Italy) dressing is a flexible polyethylene cloth coated with nanocrystalline Ag particles at a concentration between 0.69 and 1.64 mg/cm^2^.

At the Plastic Reconstructive Surgery Division of the University Hospital of Padova, Acticoat™ Flex 3 is used for burn wounds with the indication of partial-thickness burn, which needs improvement of wound-bed quality for faster recovery. Acticoat™ Flex 3 is fixed with metallic sutures after performance of surgical toilette of the wounds. The gauze dressing over the wound is moistened with distilled water and changed weekly. Patients are discharged from the hospital after taking into account their general condition; this is usually 7–10 days post-operation, and further, out-patient care is provided on a regular weekly basis. The Ag NP dressing is removed at around day 14. If the wound is recalcitrant, the patient is then subjected to further wound cleaning and autologous skin grafting. If reactivation of re-epithelialization from the edges is observed, the wound is treated with Vaseline® gauze plus Amukine Med 0.05% (a sodium hypochlorite cutaneous solution).

### 3.2. *In Vitro* Study

#### 3.2.1. Cell Cultures

**Figure 9:**
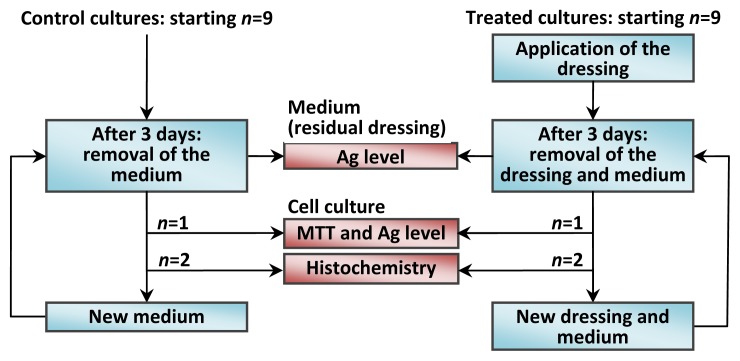
Flow chart of the experimental plan adopted for the *in vitro* study. The cycle of applications is repeated to carry out the chemical, toxicological and mitochondrial functionality determinations for 3, 6 and 9 days of cumulative duration of the treatment.

Human dermal fibroblasts were prepared according to a modified version of the Rheinwald and Green protocol [60]. After epithelial sheet dispase removal, the dermis was cut into small pieces (2–3 mm^2^), and fibroblasts were isolated by sequential digestion with 0.25% *w*/*v* trypsin for 20 min and 0.25% *w*/*v* collagenase for 4 h. These cells were then cultured with Dulbecco’s Modified Eagle Medium (DMEM), (Lonza S.r.l., Milano, Italy) supplemented with 10% Fetal Bovine Serum (FBS) (Bidachem S.p.A., Milano, Italy) and 100 units/mL penicillin and 100 μg/mL streptomycin to form complete DMEM (cDMEM). The medium was changed twice a week, and the cells were harvested by trypsin treatment. After detachment from culture plates, fibroblasts were cultured in 3D collagen-based scaffolds (MatriDerm®, Dr. Suwelack Skin & Health Care AG, Billerbeck, Germany) at a density of 1.2 × 10^5^ cells/cm^2^, obtaining a reconstructed dermal-like tissue *in vitro*. The number of the cells used to seed was derived from previous studies by Zavan and co-workers [42,43,61]. Cells were grown in the 3D scaffold for 10 days in 800 μL of cDMEM. The Ag NP-based dressing was applied above the 3D cell cultures. To guarantee experimental reproducibility, all the experiments were carried out using subsamples taken always from the same dressing and scaffold. Both the dressing and scaffold was cut with skin biopsy punches. For the dressing, a 6 mm internal diameter punch was used to obtain 40 subsamples, whereas for the scaffold, an 8 mm internal diameter punch was employed to obtain 15 subsamples. The flow chart of the experimental plan is reported in Figure 9. Every 3 days, the dressing was removed from the culture and substituted with a new one. Concurrently with dressing change, the culture medium was removed and replaced with fresh medium.

As reported in Figure 9, one set of 9 control and 9 treated cultures was created. At fixed time points (3, 6, 9 days), an MTT assay was carried out on a control and treated sample to verify mitochondrial activity. Histological analyses were performed in duplicate to verify cell distribution within the collagen-based scaffold, in the control and Ag NP treated samples. Cell culture medium and the used dressings have been collected to quantify the Ag release by ICP-MS analysis, as reported in Section 3.4.

#### 3.2.2. MTT Assay

To determine the kinetics of cell growth with or without Ag NPs, the MTT-based (methyl-thiazolyl-tetrazolium) cytotoxicity assay was performed according to the method of Denizot and Lang with minor modifications [62]. This colorimetric assay is an indirect method for assessing cell growth and proliferation. MTT gives a yellowish aqueous solution, which, on reduction with dehydrogenases or reducing agents present in metabolically active cells, yields a violet-blue water insoluble dye compound, formazan. The lipid soluble formazan is extracted with organic solvents and quantified spectrophotometrically. The amount of MTT formazan produced is directly proportional to the metabolic activity of cells.

After harvesting the culture medium, the cells were incubated for 3 h at 37 °C in 1 mL of 0.5 mg/mL MTT solution prepared in phosphate buffer saline solution (PBS). After removal of the MTT solution by pipette, 0.5 mL of 10% dimethyl sulfoxide in isopropanol (iDMSO) was added to extract the formazan in the samples for 30 min at 37 °C.

For each sample, absorbance values at 570 nm were recorded in duplicate on 200 μL aliquots deposited in microwell plates using a multilabel plate reader (Victor 3 Perkin Elmer, Milano, Italy).

The mitochondrial functionality in the Ag NP-treated cells is calculated as the ratio between the absorbance at 570 nm of the treated sample and the absorbance of a control sample expressed as a percentage.

#### 3.2.3. Morphological Analysis

Concurrently with the MTT assay, morphological analyses were carried out on the duplicate samples. After removing the Ag NP-based dressing and the culture medium, the remaining dermal-like tissue was embedded in Optimal Cutting Temperature (OCT) compound, frozen in liquid nitrogen and preserved at −80 °C until cutting. Tissue sections (>7 μm thickness) were obtained using a cryostat (CM1950, Leica, Milano, Italy) and deposited onto gelatin-coated glass slides. They were fixed with absolute acetone for 10 min at room temperature and cryopreserved at −20 °C until use. In order to visualize the cell distribution inside the scaffold and to investigate the possibility of nuclear fragmentation, the fibroblasts nuclei were stained with Hoechst H33342 fluorochrome (Sigma Aldrich, Milano, Italy, final concentration of 2 μg/mL). The samples were observed using a Zeiss Axioplan fluorescence microscope equipped with a digital camera (DC500, Leica, Milano, Italy).

In order to quantify the number of live cells and highlight the presence of apoptotic cells, a parallel set of *in vitro* experiments were carried out. Hoechst H33342 dye was added to the 3D dermal-like cell culture simultaneously with YO-PRO®-1 iodide dye (excitation wavelength 491 nm/emission wavelength 509 nm, Molecular Probes). Hoechst H33342 dye stains the nuclei in the whole population of cells, while YO-PRO®-1 stains specifically the apoptotic cells. YO-PRO®-1 is a green fluorescent probe, which can enter cells once their plasma membrane has reached a certain degree of permeability. The cell membrane during apoptosis becomes slightly permeable and YO-PRO®-1 can freely enter the cell and bind to its nucleic acids, enhancing its fluorescence intensity. The number of different cells was counted, and live cells are calculated as the difference between the number of cells stained with Hoechst H33342 and the number of apoptotic cells stained with YO-PRO®-1. Immediately after the removal of the Ag NP-based dressing from the 3D cell cultures, Hoechst 33342 and YO-PRO®-1 were added to the cell cultures. The cells were incubated at 37 °C for one hour, and then, the culture multiwell plate containing the cells was transferred to a confocal laser scanning microscope to monitor YO-PRO®-1 and Hoechst fluorescence.

A fluorescence confocal laser scanning microscope (Axiovert 100M, Zeiss, Germany) with a 10× magnification objective was used for the detection of Hoechst H33342 and YO-PRO®-1 stained cells. The fluorescent dye, YO-PRO®-1, was excited with a 25 mW Argon laser at 488 nm. Emission was recorded above 510 nm. The Hoechst H33342 fluorescence was detected at 460 nm after excitation at 346 nm. The microscope was equipped with a motorized stage, and the LSM 510 (Zeiss) software enabled memorization of stage positions. For each sample, images were taken at the preset stage positions at various depths. The count of the live cells in the sample is obtained as the sum of the live cells at various depths at each position.

### 3.3. *In Vivo* Study

#### 3.3.1. Human Skin Samples

Patients were eligible for the study if recruited <24 h post-burn injury and were affected by partial-thickness burns. Patients were excluded if they were affected by full thickness burns or had a compromised immune system or were known to be hypersensitive to silver and its compounds. Patients were also excluded in case of comorbidity (e.g., diabetes, cardiac or renal disease), chemical or electrical burns, multiple trauma or were aged <5 or >60. Skin biopsies were obtained from a set of eligible patients who gave consent for taking biopsy materials for scientific purposes, and the study was performed in compliance with the Declaration of Helsinki ethical guidelines.

**Figure 10:**
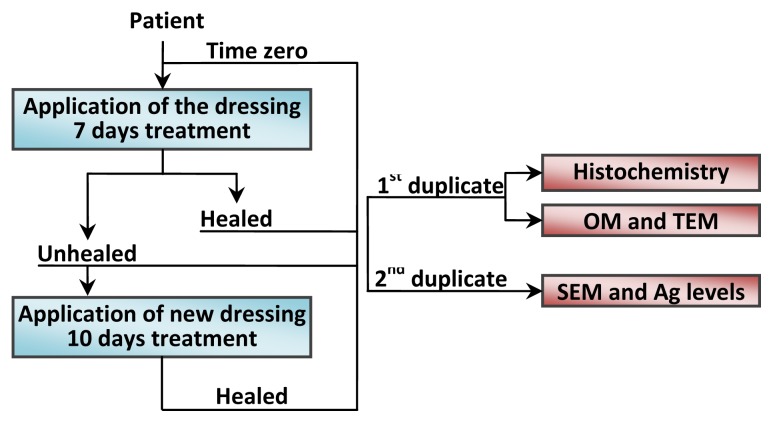
Flow chart of the experimental plan adopted for the *in vivo* study.

Biopsies were collected by using punches of 4 mm inner diameter × 7 mm depth, according to the experimental plan represented in Figure 10, from 1 patient. Duplicate samples were collected from the same patient before application of the dressing (time zero). After seven days of treatment at dressing removal, two more duplicates were taken, one from the healed area and another from an unhealed zone. After 10 more days of treatment with a new dressing, another duplicate sample was taken from the newly healed area. For each sample, the first duplicate was preserved in formalin and then divided vertically into two portions: one was used directly for histological analysis after hematoxylin and eosin staining, while the other part was transferred into a 2.5% glutaraldehyde solution and, subsequently, prepared for analysis by transmission electron microscopy (TEM). The second duplicate was immediately frozen and then used for the environmental scanning electron microscopy (ESEM) and chemical analysis, as reported in Section 3.4.

#### 3.3.2. Optical Microscopy, TEM and ESEM

The samples preserved in formalin were used for histological analyses to evaluate the tissue structure during the course of the healing process. They were then paraffin-embedded, stained with hematoxylin and eosin and observed using optical microscopy (OM).

To select the areas with the most altered cellular morphology and, hence, potential Ag localization, a preliminary morphological characterization of the other vertical portion was carried out on semi-thin sections after toluidine blue staining (1% toluidine blue 1% borax for OM). The selected areas were then re-sampled to provide information on the distribution of Ag NPs at the sub-cellular level. This is done by fixing in a 2.5% glutaraldehyde/0.1 M sodium cacodylate buffer overnight at 4 °C. The samples were then treated with 1% OsO_4_/0.1 M sodium cacodylate buffer and dehydrated using ethanol solutions of increasing concentrations before embedding in EPON™ epoxy resins. Ultrathin sections (ultramicrotome, LKB, Stockholm, Sweden) were obtained and treated with 1% uranyl acetate and 1% lead citrate. The samples were analyzed by TEM (Electronic Microscopy Service, Department of Biology, University of Padova, Padua, Italy) using a Tecnai G12 electron microscope (FEI, acceleration voltage 100 kV). The image acquisition system consisted of a video camera, TIETZ (Tietz Video and Image Processing Systems GmbH, Gauting, Germany), and the TIA FEI Imaging Software (FEI Company, Hillsboro, OR, USA).

The ESEM analysis was carried out at the Interdepartmental Service Center C.U.G.A.S. (University of Padova) using a Quanta 200 (FEI company, Hillsboro, OR, USA) instrument equipped with an energy dispersive X-ray (EDAX Inc; Mahwah, NJ, USA) detector. Samples were dried in a thermostatted oven at 37 °C for 24 h prior to analysis. The samples were analyzed directly after placing on a vitreous carbon planchet, without metallization. Backscattered electron images were collected at 25 kV. Representative energy-dispersive X-ray spectroscopy (EDS) spectra were collected in potentially interesting sites to obtain a qualitative indication of the elemental composition of the surface and particles identified in the samples.

### 3.4. Determination of Ag Levels by ICP-MS

The concentration of Ag in all samples (dressing, culture medium, cell culture extracts and *in vivo* collected skin) were determined by inductively coupled plasma-quadrupole-mass spectrometry (ICP-QMS) using a model 7500cx instrument from Agilent Technologies (Tokyo, Japan). The main instrumental parameters are reported in Table 3, whilst sample preparation is briefly outlined below. All reagents were purchased from Sigma-Aldrich, Milan, Italy.

New (unused) and residual dressings (collected after the *in vitro* application) were mineralized by acid digestion in an Ethos1 (Milestone) microwave oven. The digestion was carried out in Teflon vessels following a two step program by adding to ~2 mg of the samples 10 mL of concentrated HNO_3_ (1st step at 200 °C, 50 min) and then 2 mL H_2_O_2_ 30% *w*/*w* (2nd step at 200 °C, 10 min). The digests of the residual dressings were centrifuged at 3000 rpm for 10 min to separate the AgCl precipitate formed, due to the presence of Cl^−^ in the cDMEM. The supernatant was collected and directly diluted in NH_4_OH 2.8% *w*/*w*, while the precipitate was dissolved in 1.5 mL of concentrated NH_4_OH (28% *w*/*w*) and then diluted in NH_4_OH 2.8% *w*/*w* for ICP-QMS analysis.

The Ag concentration was also determined in cDMEM (~400 μL) collected from the cell culture (both untreated and treated with Ag NPs) used for MTT and histological analyses. The solutions were directly diluted in NH_4_OH 2.8%, tetramethylammonium hydroxide (TMAH) 0.1% and Triton-X 0.1% w/w and analyzed for total Ag by ICP-QMS, as above.

The concentration of Ag in the cell culture (and the scaffold) was indirectly estimated by the difference between the level in the new dressing and the combined levels found in the residual dressing and in the medium. To validate the estimates, they were compared with the direct measurement of the Ag concentration in the cell cultures (plus scaffold) previously used for the MTT assay (the only non-destructive analysis). The Ag concentration in the cell culture and underlying scaffold was obtained as the sum of the concentrations measured in the fraction collected after MTT incubation and in the reunified iDMSO fractions after absorbance measurement. The iDMSO fraction was treated with 1.5 mL of NH_4_OH 28% *w*/*w* to solubilize any Ag attached to the scaffold; the sample was then centrifuged at 3000 rpm for 10 min. The supernatant was diluted to a final volume of 15 mL in TMAH 0.1% and Triton-X 0.1%. The MTT fraction was first diluted (1 + 14) in a solution containing NH_4_OH 2.8% TMAH 0.1% and Triton-X 0.1%. This solution was then used to dilute both fractions (200 times) prior to ICP-QMS analysis. A parallel control experiment at the 3 day time point in the absence of cells was carried out to quantify the partitioning of silver between the fractions described above and the scaffold. In this experiment, after extraction with iDMSO, 500 μL of concentrated ammonium hydroxide solution (28%, as sold by Sigma Aldrich) was added to the vial containing the collagen-based matrix and the iDMSO solution. To ensure that all the silver attached to the surface of the scaffold was removed, the vial was vigorously shaken for 1 min at room temperature. The solution was removed by pipette, and the collagen-based matrix was washed twice with 500 μL concentrated ammonium hydroxide solution to recover the amount of surface bound Ag from the scaffold and to solubilize any insoluble silver forms present (in particular, AgCl or silver bound to insoluble proteins, as reported in Rigo *et al.*[47]). All the collected fractions were reunified within the iDMSO fraction and analyzed by ICP-MS. The remaining scaffold was mineralized in 500 μL of concentrated TMAH (25% *w*/*v* in water) and was then analyzed by ICP-MS to quantify the amount of Ag trapped in the scaffold.

Once the ESEM-EDS analysis was completed, the biopsies collected *in vivo* were cut into transverse sections of approximately 2 mg weight. Concentrated TMAH solution (100 μL) was added to each section, and alkaline digestion was carried out overnight in a thermostatic bath at 60 °C. The digests were diluted 200-fold in 0.1% Triton X-100 and NH_4_OH 2.8% *w*/*w* solution and analyzed for total Ag concentration by ICP-QMS, as reported above.

## 4. Conclusions

In this pilot study, *in vitro* results indicate the safety of Acticoat™ Flex 3. It seems that Ag NPs may reduce mitochondrial functionality, but this probably occurs temporarily. As indicated by our results, the reduction in mitochondrial activity does not affect cell viability. The *in vivo* study, although limited in scope, since it was conducted on a single patient, by taking a biopsy from an unhealed area and a healed area of the same wound, seems to support the absence of toxicity: in the healed skin, no signs of apoptosis or necrosis were observed, despite the presence of a great quantity of Ag NPs in the cytoplasm of the fibroblasts. We observed that a Ag NP-based dressing does not create an obstacle to the recovery of severe partial thickness burns. After application for an extended period (17 days), the organized skin structure (dermis and epidermis) was re-established in a previously unhealed part of the wound. In this pilot study, we have also demonstrated that the application of Ag NP-based dressings, even for a prolonged time, does not seem to negatively affect the proliferation of fibroblasts and keratinocytes, leading to the restoration of normal skin.

## Figures and Tables

**Table 1:** The results for three separate applications of Ag to the culture, expressed as μg of Ag per cm^2^ (μg cm^−2^) of culture covered dressing. Applied Ag levels are assumed to be equal, based on analysis of the new unused dressing. Silver concentrations in the dressings and the medium were determined by ICP-MS. The Ag concentration in the 3D culture was obtained by difference.

		Ag (μg cm^−2^)			
		
Days	Sample size	Total applied	Dressing	Medium	Culture *
1–3	*n* = 9	821	767 ± 4	37 ± 5	18 ± 3
3–6	*n* = 6	821	765 ± 3	31 ± 3	25 ± 2
6–9	*n* = 3	821	765 ± 4	32 ± 2	25 ± 3

Note:

* calculated as the mean of the differences.

**Table 2:** Cumulative concentration of Ag (μg cm^−2^) in the cell culture (*n* = 1) and control experiment (*n* = 3) with a comparison of the values estimated by difference with the sum of the direct measurements in the MTT and dimethyl sulfoxide in isopropanol (iDMSO) fractions. TMAH: tetramethylammonium hydroxide.

Days	Estimated	MTT + iDMSO	MTT	iDMSO	TMAH
3	16.5	16.3	5.8	10.5	-
6	40.9	30.9	8.0	22.9	-
9	62.9	46.5	10.3	36.2	-

**Control experiment in absence of cells*n*= 3 replicates ± 1 SD**					

3	-	12.5 ± 5.1	8.9 ± 3.7	3.3 ± 1.8	0.7 ± 0.3

**Table 3:** Inductively coupled plasma-quadrupole-mass spectrometry (ICP-QMS) operating conditions.

RF power	1500 W
Plasma gas flow rate	15 L min^−1^
Carrier gas flow rate	1.2 mL min^−1^
Nebulizer	V-groove
Ions lens setting	Optimized daily for best sensitivity using a 10 ng mL^−1^ Ag in NH_4_OH 2.8% *w*/*w* solution
Monitored masses	107, 109
Points per peak	3
Acquisition time per point	1 s
Replicates	5
